# Evaluation of Neoproterozoic source rock potential in SE Pakistan and adjacent Bikaner–Nagaur Basin India

**DOI:** 10.1038/s41598-022-14831-5

**Published:** 2022-06-30

**Authors:** Qamar Yasin, Syrine Baklouti, Ghulam Mohyuddin Sohail, Muhammad Asif, Gong Xufei

**Affiliations:** 1 College of Energy and Mining Engineering, Shandong University of Science and Technology, Qingdao, 266590 China; 2 Institute of Geophysics, Polish Academy of Sciences, Warszawa, 01-452 Poland; 3 Department of Geological and Environmental Engineering, National School of Engineers of Sfax, Sfax, Tunisia; 4 Department of Geological Engineering, University of Engineering and Technology, Lahore, Pakistan; 5 Department of Civil, Geological and Environmental Engineering, University of Saskatchewan, Saskatoon, S7N 5C5 Canada; 6 ExxonMobil Upstream Research Company, 22777 Springwoods Village Pkwy, Spring, TX 77389 USA

**Keywords:** Solid Earth sciences, Geochemistry, Geology, Geophysics, Tectonics

## Abstract

Discoveries of heavy crude oil in the Neoproterozoic rocks (Infracambrian rock sequence) from the Bikaner-Nagaur Basin of India emphasize the significance of studying and exploring the Neoproterozoic source rock potential in the southeastern part of Pakistan. This study evaluates the potential of the source rock in the Infracambrian rock sequence (Salt Range Formation) based on surface geochemical surveys, Rock–Eval pyrolysis, source biomarkers, geophysical characterization, and seismic inversion using machine learning for maturity index estimation. Core samples of Infracambrian rock were extracted for Rock–Eval pyrolysis and biomarker characterization. Additionally, 81 geomicrobial soil and gas samples were collected from the surface to explore the petroleum system and potential source rocks in the subsurface. Advanced interpretation techniques were used to investigate the origin and concentration of hydrocarbon gases at the surface, including Rock–Eval pyrolysis, thermal maturity, source biomarkers, and the environment of deposition of organic matter. The results show that the investigated samples are characterized by restricted marine clay devoid of sedimentary carbonate facies with thermal maturity in the early stage of the oil generation window. The seismic inverted maturity index profile demonstrates a reasonable correlation of thermal maturity with the biomarkers and Rock–Eval pyrolysis. Further scrutiny of the surface geochemical samples confirms the presence of higher concentrations of thermogenic C_2_–C_4_ hydrocarbons in the vicinity of anticlinal structures, suggesting the existence of an effective migration path along deep-seated faults to the surface. This study concludes that the Infracambrian rocks on the eastern flank of Pakistan are thicker, thermally mature, and have deep-seated structural closures, indicating a greater probability of heavy and light oil in this area than in the Bikaner–Nagaur Basin, India.

## Introduction

Neoproterozoic rocks are among the oldest petroleum-producing rock units in the world, and several successful discoveries have been reported from these sequences in the Salt Basins of Oman, India, Morocco, Venezuela, China, and Siberia^1–7^. Although proven hydrocarbon plays exist in many regions of the world, the Infracambrian rock sequence has received less attention for petroleum exploration than younger sedimentary strata. Infracambrian rocks have been intensively investigated and generate economic hydrocarbons only in Russia, Oman, and China. The discovery of the world's oldest oil from these countries illustrates the generation and migration of Proterozoic petroleum, implying that the hydrocarbon potential of certain unmetamorphosed Infracambrian rock sequences in various parts of the world should be carefully investigated^6^.

The paleogeographic plate reconstructions for the Infracambrian and Cambrian periods show restricted marine evaporites in the Salt Range Formation (Pakistan), Bikaner–Nagaur Basin (NW India), and Huqf Basin (southern Oman). They were all close together on a broad carbonate shelf on the northern margin of Gondwana during the Infracambrian and Cambrian periods^8–11^. Additionally, the age and lithologies of the Salt Range Formation and the Marwar Group of Bikaner-Nagaur (BK-N) are similar to those of the Huqf Group (southern Oman), which has proven to be the source rock^2^. In the BK-N Basin in India, nonbiodegradable heavy oil reserves have been discovered in the Infracambrian Jodhpur sandstone, the Bilara dolostone, and the thin siltstone layers in the Hanseran evaporite with porosities ranging from 16–25%, 7–15%, and 3–12%, respectively^5,12^. Heavy oil is nonbiodegradable, and biomarker ratios support the presence of crude oil from the source rock in the early oil generation window^2,13–16^. In the BK–N Basin, the biomarker study from the Baghewala-1 well reveals that mature hydrocarbons have been generated from a deeper buried source rock^2^. Several authors reported that the same quantities of petroleum could be generated from the Infracambrian source rock of the Salt Range (SR) Formation in southeastern (SE) Pakistan, where Infracambrian source rock beds are much deeper and thicker than those in the BK–N Basin^2,9,12^.

Biomarkers are organic compounds genetically related to their biological precursors and can be studied to establish the origin and evolution of organic matter (OM) during postdepositional processes^17^. Baghewala-1 oil was derived from Infracambrian rocks that contained algal and microbial OM that was deposited under anoxic marine conditions^2^. These findings showed that the organic-rich laminated dolomites in the Infracambrian rocks were developed locally and stimulated further investigation of the biomarker properties of the Infracambrian SR Formation.

Surface geochemical surveys allow rapid and low-cost preliminary identification of hydrocarbon-bearing regions from hydrocarbon microseepages^18–21^. The underlying concept of surface geochemical prospecting is that faults and fractures provide pathways for light gaseous hydrocarbon migration from subsurface geological formations to the surface or shallow environment by diffusion and effusion, resulting in anomalies of surface hydrocarbon gases^22,23^. The existence of all hydrocarbon gas components, such as methane, ethane, propane, butane, and pentane, in the surface soils indicates the possibility of the presence of an active petroleum system in the subsurface^24^. Additionally, anomalous concentrations of thermogenic C_2_–C_4_ hydrocarbons at the surface confirm the occurrence of mature source rock in the basin^21^.

In recent years, the Infracambrian play in SE Pakistan has been explored to assess the hydrocarbon potential by geological modeling or seismic data characterization^9,12,25–27^. However, previous attempts have focused only on the distribution of Infracambrian sources and reservoir rocks in SE Pakistan. Nevertheless, no study has directly investigated the potential and maturity of Infracambrian source rocks. In this study, we evaluate the distribution and potential of Infracambrian source rocks based on a combination of surface geochemical prospecting, biomarker parameters, geophysical characterization, and seismic inversion using supervised machine learning algorithms, i.e., particle swarm optimization (PSO) and multilayer linear calculator (MLC), to transform the seismic sections into maturity index (MI) profiles. This is the first time we have investigated the area utilizing a combination of all of these techniques. Furthermore, we correlated the origin of surface microbial gases with gravity modeling and seismic data interpretation. Additionally, the stratigraphic succession of the study area was compared with that of the BK-N Basin to understand the Infracambrian stratigraphic variation, thickness, and burial depth of source rocks.

## Regional geological setting and stratigraphy

### Geological setting

**Figure 1:**
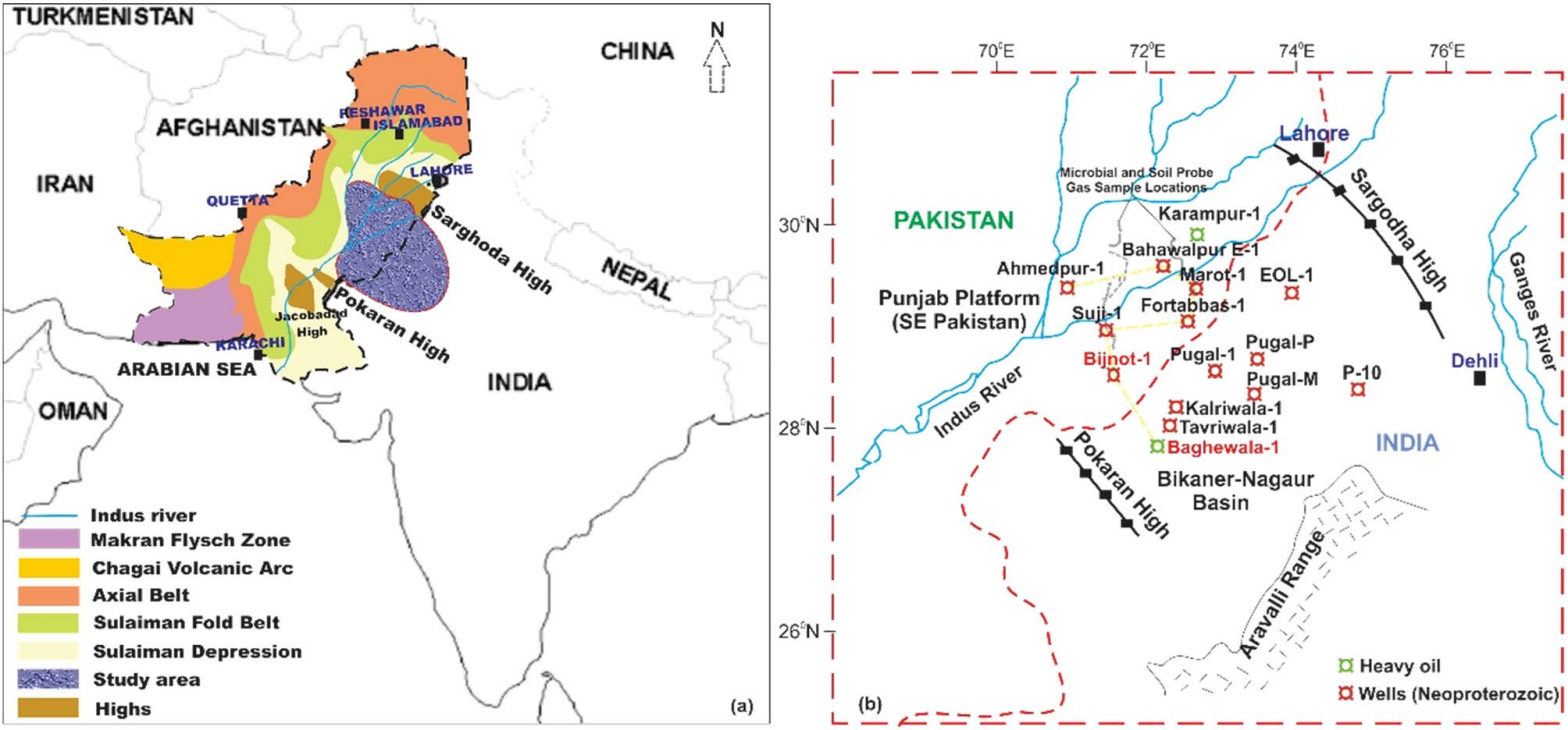
(**a**) Tectonic map of Pakistan indicating structural features and locations of the study area, and (**b**) a map showing wells located in SE Pakistan and Bikaner–Nagaur Basin India^2,32^. The samples were collected from the wells labeled in red font. A yellow line indicates a stratigraphic cross-section in Fig. 3. CorelDRAW X5 was used to create the illustration (www.coreldraw.com).

The study area is located in the eastern segment of the central region of the Lower Indus Basin, which is divided into the Punjab Platform and the Suleiman Fold Belts, and the BK-N Basin in NW India is bounded by the Sargodha High to the north and the Pokaran High to the south (Fig. 1a,b). The entire area is covered by Quaternary alluvium that extends westward along with the Suleiman Fold Belts^28,29^. In SE Pakistan, the Punjab Platform covers approximately 58,000 km^2^ and extends to the BK-N Basin eastwards in Rajasthan, India (Fig. 1a). Seven exploratory wells have been drilled in the Late Neoproterozoic-Early Cambrian rocks in SE Pakistan, where heavy oil was observed in the SR Formation from the Karampur-1 well (Fig. 1b). This heavy oil geochemically resembled the heavy nonbiodegradable oil from the Jodhpur sandstone, the Bilara dolostone, and the Hanseran evaporite of the Baghewala-1 well in the BK–N Basin^2,15,16^. Similar geochemistry of heavy oil has been reported for the carbonate-evaporite facies of the Huqf Group (Infracambrian), located more than 2000 km to the SW, along the eastern flank of southern Oman^2,12,25,27^. These Infracambrian rocks were once within or near the western edge of Gondwanaland, which was marked by a subsiding rift system, and today, these rocks crop out to the west of the Aravalli ranges in the BK-N Basin and extend toward northern and northwestern Pakistan^1,2,9^. The exposures of the Aravalli Range in the northwestern Indian Shield allowed the development of sedimentary basins along its flanks (Fig. 1b). The movement of the tectonic plates is responsible for the Precambrian rocks' deformation and caused several subsided and uplifted blocks. Tectonic subsidence resulted in the development of the BK–N Basin, which is related to the Kirana–Tosham Basin^9^. The local highs that existed in the study area were inferred from Early Infracambrian times that framed the basin configuration as more complicated. The seismic data show the eastward progradation of seismic reflections that could be interpreted as Paleozoic and Mesozoic nonorogenic movements (not severely deformed) in the central region of the Indus Basin^25,29^. The bedding is dominantly oriented to the west, i.e., nonorogenic movements tilted the central region of the basin eastward in the Paleozoic. Moreover, in the Mesozoic, it was inclined to the west. Thus, it is assumed that the Neoproterozoic deposition was primarily controlled by horst and graben structures, possibly accompanied by the breakup of Pannotia^9,11^.

The Infracambrian SR Formation is exposed in the northeastern part of Pakistan (Potwar Plateau)^9,25^. Exposures of Precambrian rocks occur as many hillocks (volcanic and volcaniclastics) that are located in the Kirana Hills (Hachi volcanic) in the Sargodha–Shahkot–Sangla–Chiniot region of SE Pakistan^30,31^. These crystalline rocks, with or without Phanerozoic cover, lie at the bottom of the Indus Basin^9^. These Hachi volcanic rocks are analogous to the Infracambrian rocks in Nagar Parkar (Tosham-Malani volcanic India).

### Lithostratigraphy

**Figure 2:**
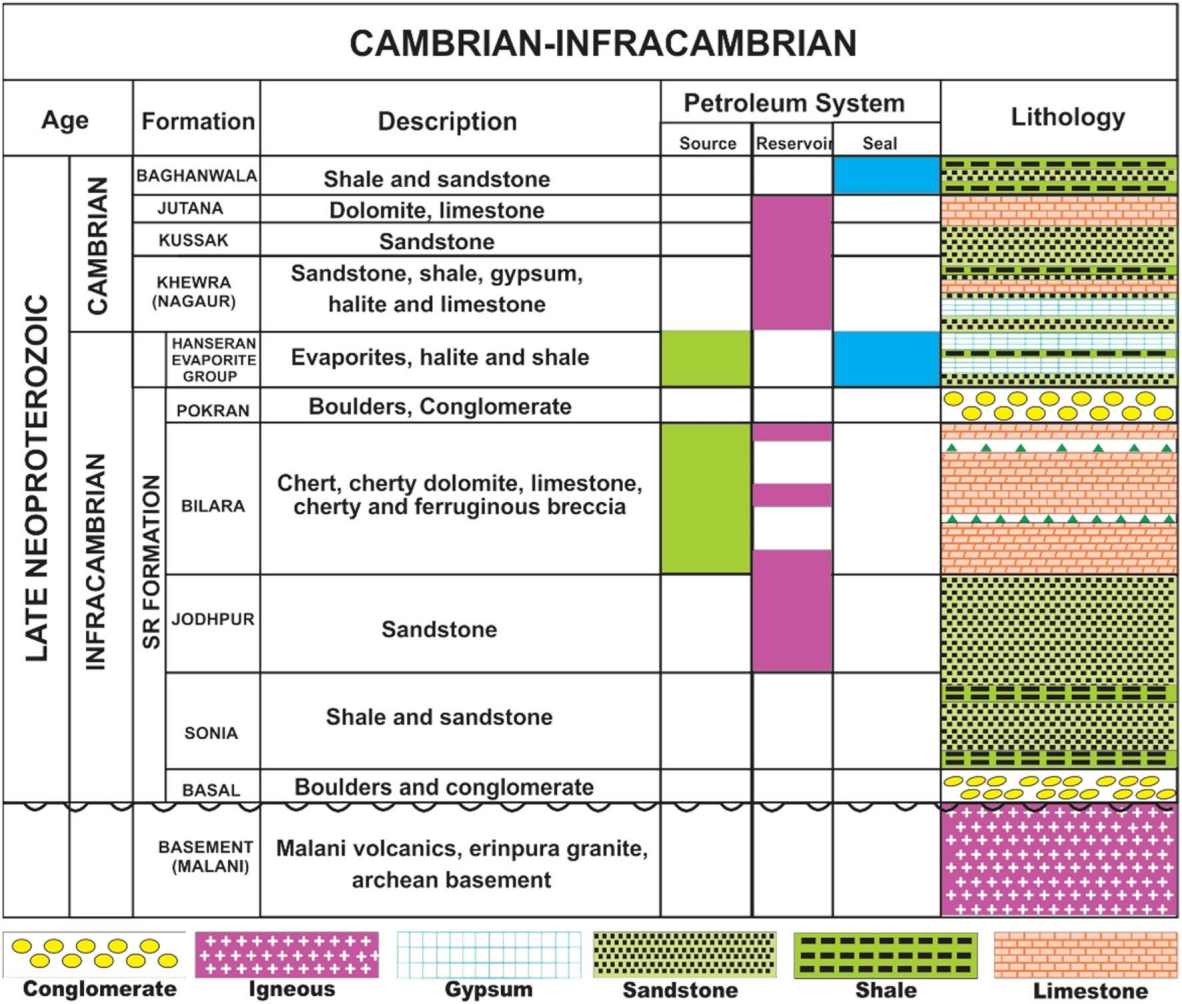
Stratigraphic column and petroleum system of the Cambrian–Infracambrian Salt Range Formation in the SE Pakistan Bikaner–Nagaur Basin^10^.

The lithostratigraphy of the study area was investigated using outcrops and well log data from the BK-N Basin India and SE Pakistan. The lithostratigraphy of the BK-N Basin is dominantly composed of nearly 1000 m of shales, sandstones, carbonates, and evaporites (Fig. 2). This lithology is similar to that of the Potwar subbasin (upper Indus Basin)^12,25^. The observed sedimentary succession indicated several regional unconformities, erosion, and hiatuses in the study area. The Infracambrian Salt Range (SR) Formation unconformably overlies a Precambrian basement^33^. The maximum thickness of the SR Formation is 906 m.

Four stratigraphic units of the SR Formation are identified in the study area equivalent to the Infracambrian rocks in the BK-N Basin. The lithostratigraphy of the SR Formation is based on the depositional environment and lithological information from the Suji-1, Marot-1, Karampur-1, and Bahawalpur East-1 wells^25,30,34^. The SR Formation is subdivided, from oldest to youngest, into four formations: Sonia, Jodhpur, Bilara, and Hanseran (Fig. 2). The Hachi volcanic suite (equivalent to the Tosham Malani igneous suite) forms the basement. Both the Hachi and Tosham Malani volcanic rocks unconformably underlie the Basal conglomerates, which are composed of dolomites and volcanic clastics. The clastic sediments of the Jodhpur Formation form the primary potential reservoir in the basin and can be classified into Sonia shale and sandstone members. The Bilara–Hanseran sequence lies conformably over the Jodhpur Formation. Bilara–Hanseran acts as a source rock with a total organic carbon (TOC) ranging from 5 to 6%^35^. The calcareous Bilara Group comprises dolomite, limestone, stromatolitic limestone, and occasional clay beds. The Hanseran Evaporite Group comprises halite, anhydrite, dolomite, and reddish sandstone with thin shale beds. The Bilara-Hanseran sequence has a total estimated thickness of approximately 200 m^35^. A comparison of the Infracambrian and Cambrian successions between the BK-N Basin and SE Pakistan is described in Table 1.

**Table 1:** Maximum thickness and petroleum system of the Cambrian-Infracambrian succession in SE Pakistan and the Bikaner-Nagaur Basin^10^.

Age	BK-N Basin India	Thickness (m)	SE Pakistan	Thickness (m)	Petroleum system
Cambrian	Upper carbonate	230	Jutana Dolomite	12–112	
			Kussak Formation	93–217	
	Nagaur Formation	180	Khewra Sandstone	101–152	
Infracambrian	Hanseran Evaporite	170	Salt, marl, gypsum, and dolomite (SR Formation)	140–1143	Source, reservoir, seal, Source
	Bilara Dolomite	40			
	Jodhpur Formation	50	Conglomerate, shale (Sharaban Formation) Hadda Quartzite, quartzite (Asianwala Formation), phyllite (Taguwali Formation)	2300	Reservoir
Precambrian	Volcanic rocks Basement (Malani Suite)	+ 150	Hachi Volcanics Basement		

## Structural style

**Figure 3:**
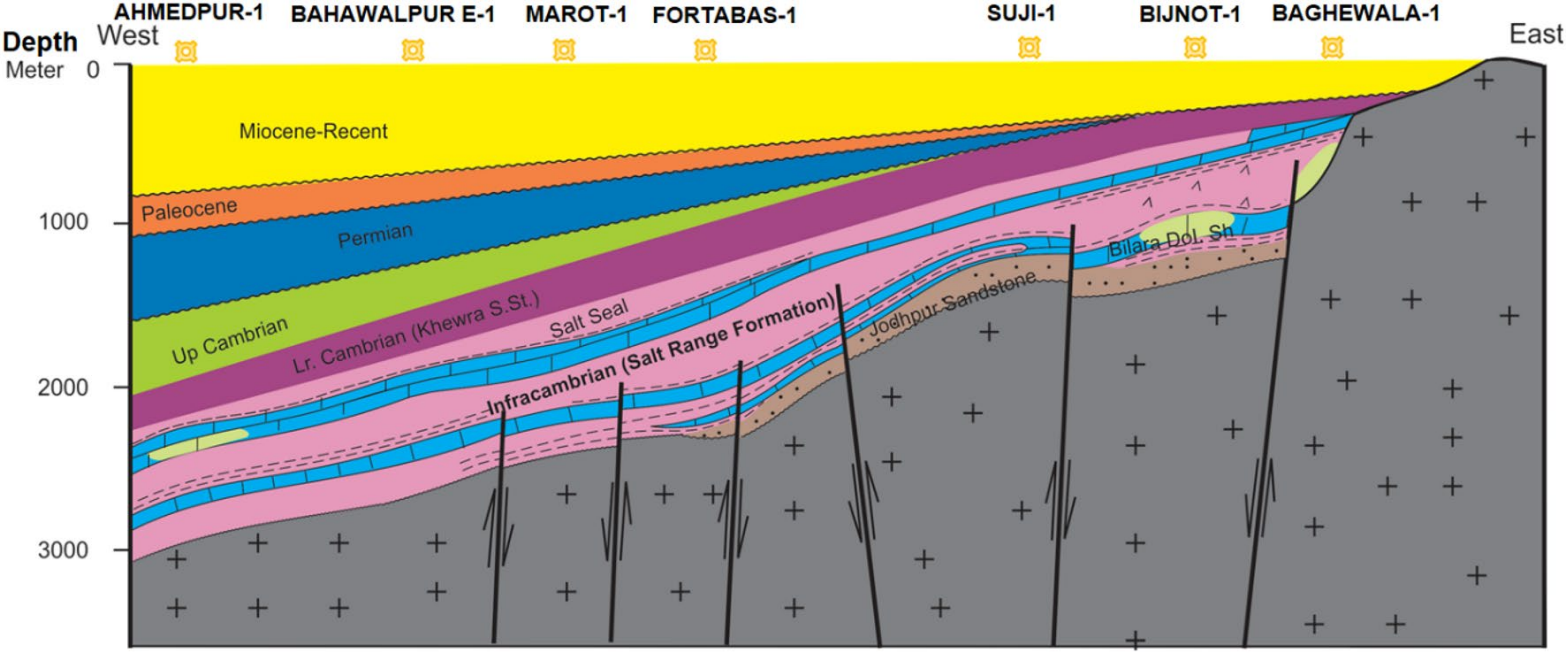
Regional schematic stratigraphic cross-section and structural interpretation along with the SE Pakistan and part of Bikaner–Nagaur Basin India. CorelDRAW X5 was used to create the illustration (www.coreldraw.com).

The regional schematic section of structural interpretation from the basement to the Eocene based on a cross-section across the wells (Fig. 1b) of Ahmedpur-1, Bahawalpur-1, Marot-1, Fort Abbas-1, Suji-1, Bijnot-1, and Baghewala-1 in SE Pakistan and BK–N Basin India is shown in Fig. 3. The correlation of these exploratory wells identifies the Infracambrian rock sequence (SR Formation) at depths varying from 1100 to 2600 m (NE to SW). It highlights several unconformities in the stratigraphic succession. From the figure, we observe that the Miocene, Paleocene, Permian, and Cambrian sediments gradually thin toward the east, contrasting with the Infracambrian deposits. The lithological log correlation classifies the Infracambrian rock into four lithofacies, i.e., shale, sandstone, dolomite, and evaporites^36^.

The basement rocks in the Karampur-1, Marot-1, Fort Abbas-1, Suji-1, Bijnot-1, and Baghewala-1 (India) wells are composed of rhyolite, weathered tuff, ash, and brittle clay with interbedded flows of basalt and trachyte. At approximately 1000 Ma, a mantle plume and rifting initiated by Infracambrian time generated widespread igneous activity during the post-Aravalli period. This wide variation in volcanic activity suggests extensional tectonics followed by deposition in the BK-N and Indus Basins.

From the rifting to the collision and underthrusting of the Indian Plate during the Infracambrian to Mesozoic, the basin is associated with deep-seated normal faults, salt-induced structures, horst and graben structural highs and lows (Fig. 3).

## Materials and methods

### Data description

Logs and core data, including well cuttings from nine wells, as shown in Table 2, were used for estimating the total organic carbon (TOC), Rock–Eval pyrolysis, biomarkers, mineralogy, saturation, porosity, and permeability in the Infracambrian to Permian rocks. Additionally, surface geomicrobial soil and gas samples were investigated to evaluate the petroleum potential of source rocks in the subsurface. Moreover, 1000 km 2D seismic data were interpreted to mark the stratigraphic horizons and structural style, focusing mainly on the top and bottom reflectors of the Infracambrian–Cambrian rocks.

**Table 2:** Summary of the exploratory wells drilled through the Infracambrian to Permian play in SE Pakistan and Bikaner–Nagaur Basin India.

Exploratory well	Time	Depth (m)	Drilled up to	Target reservoir	Comments
Karampur-1	1959	3034	Basement	Base Permian	Heavy asphaltic oil
Bahawalpur East-1	1980	3024	Basement	Base Permian	Gas shows
Marot-1	1981	2596	Basement	Base Permian	Fluorescence
Baghewala-1 (India)	1991	1570	Basement	Infracambrian	Oil discovery
Pugal-1	1991	0678	Precambrian	Infracambrian	Heavy oil
Fort Abbas-1	1994	1651	Precambrian	Base Permian	Abandoned
Bijnot-1	1996	1914	Basement	Infracambrian	Good oil shows
Suji-1	2000	2628	Basement	Infracambrian	Low maturity
Maharvi-1	2007	2980	Basement	Infracambrian	Low maturity

### Methodology

**Figure 4:**
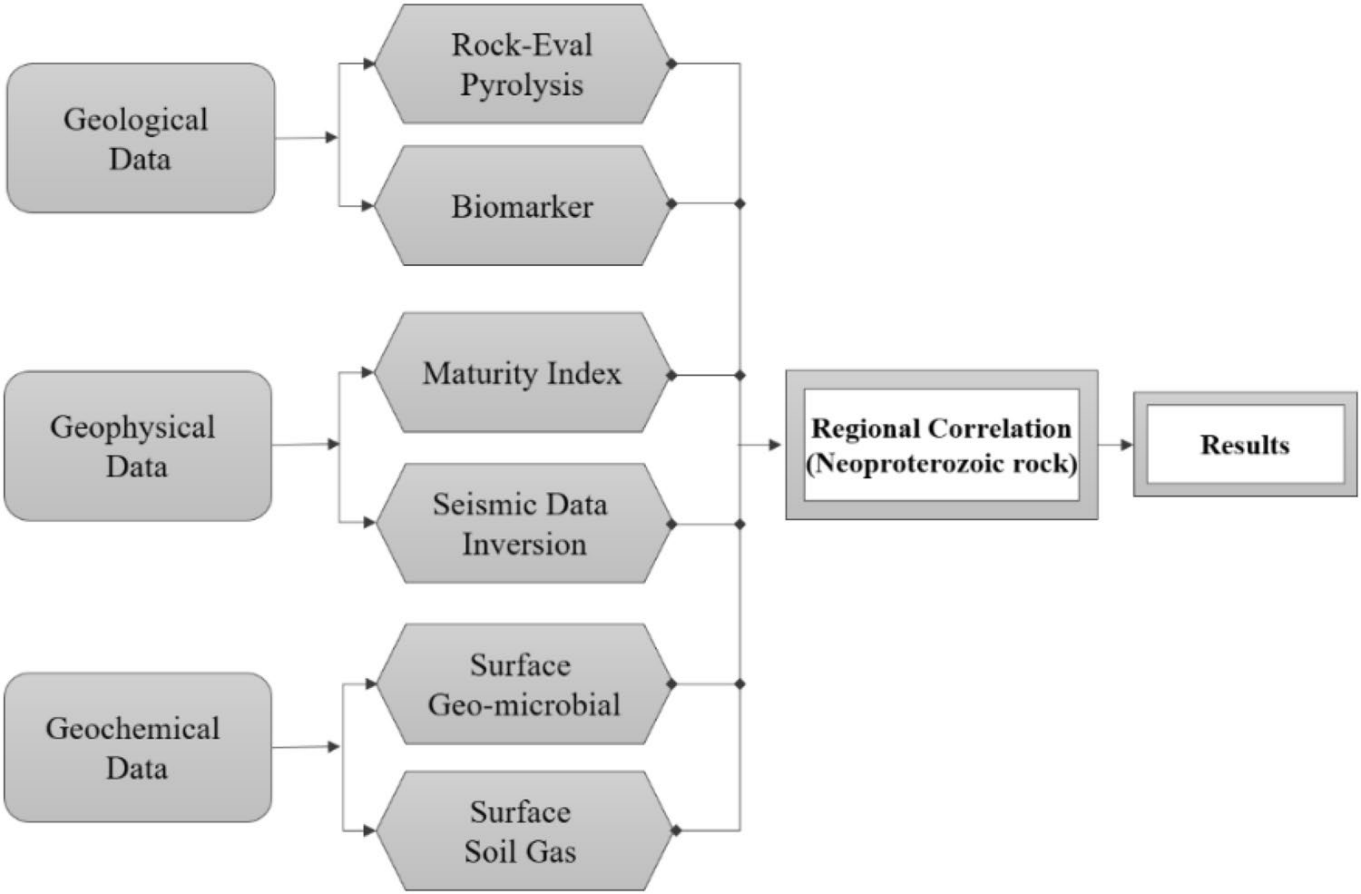
Flow chart of the methodology.

The following sequence was adopted to achieve the study's objectives (see Fig. 4).

#### Rock–Eval pyrolysis

Rock–Eval pyrolysis was used to identify the maturity of OM and hydrocarbon generation potential of the Infracambrian rock. Four basic parameters, the oxygen index (OI), production index (PI), hydrogen index (HI), and maximum temperature (T_max_), were obtained by pyrolysis. The chosen samples (Table 3) were measured using a Vinci Rock–Eval 6 apparatus following previously reported procedures^37–39^. Ten well cuttings samples from Bijnot-1 were investigated to measure the OI, HI, PI, T_max_, and TOC. LECO's CS230-series carbon and sulfur determinator was used to measure the TOC content of the well cutting samples following the formal standards process (CNS GB/T19145-2003).

**Table 3:** Rock–Eval pyrolysis of Bijnot-1 well cuttings from the SR Formation.

Sample	Depth (m)	S_1_ (mg HC/g Rock)	S_2_ (mg HC/g Rock)	S_3_ (mg HC/g Rock)	HI (mg HC/g TOC)	OI (mg CO_2_/g TOC)	PI	T_max_ (°C)	TOC (wt%)
BJ-01	1622	0.73	1.01	1.32	127	165	0.42	424	0.81
BJ-02	1634	0.74	0.95	1.45	122	178	0.43	420	0.82
BJ-04	1642	0.81	10.55	2.11	451	94	0.08	425	2.41
BJ-05	1662	0.25	3.10	1.51	342	166	0.08	432	0.92
BJ-06	1682	0.22	0.81	1.36	203	338	0.20	432	0.61
BJ-08	1700	0.32	0.63	1.50	141	328	0.32	432	0.65
BJ-09	1742	0.36	0.42	1.41	104	338	0.44	433	0.62
BJ-10	1762	0.52	0.58	1.64	113	322	0.46	424	0.51
BJ-12	1794	1.41	2.40	1.90	212	170	0.38	429	1.32

Pyrolysis generates peaks that are transformed into measurable surfaces of S1, S2, and S3. The P1 peak corresponds to thermovaporizable hydrocarbons released at 300 °C, whereas the P2 peaks refer to pyrolyzable hydrocarbons produced by cracking kerogen over 300 °C. The pyrolysis temperatures are calibrated to ramp up at an average of 25 °C/minute for 20 min. T_max_ (°C) is the temperature at the top of the P2 peak (maximum hydrocarbon genesis). Organic CO_2_ determination is theoretically possible if trapping is interrupted when the oven temperature exceeds 400 °C.

#### Biomarker analysis

Ten well cutting samples were also extracted for sedimentary OM characterization. Extracts were separated by liquid chromatography into aliphatic and aromatic fractions of hydrocarbons and further analyzed by gas chromatography–mass spectrometry (GC–MS). Aliphatic and aromatic biomarkers were analyzed to evaluate the organic matter source facies, thermal maturity, and deposition environment. The methods, techniques, and guidelines that were used are similar to those reported in the literature^40–42^.

#### Maturity index (MI) calculation

Zhao et al.^43^ developed a relationship between MI and T_max_ to determine the kerogen type. They established two baselines, i.e., MI < 5 referring to the oil with some dissolved gases and MI > 7 indicating dry gases without any condensate. The values of MI between 5 and 6 indicate oil (or condensate) and wet gas.

The bulk density, neutron porosity, shear and compressional sonic and deep resistivity logs of the Bijnot well, which are sensitive to maturity variations, were utilized to compute the MI in the Infracambrian rock by employing Eq. (1). In this equation, Zhao et al.^43^ evaluated the total and effective porosities of the formation from the core samples and correlated it with porosity calculated from the density log in the same interval to determine the threshold values of MI.

In this study, data of samples with log density porosity greater than 9% and water saturation less than 75% were included in the calculations of MI by the following equation:

1 $$MI = \,\,\sum\limits_{i = 1}^{N} {\left( {\frac{N}{{\varphi_{n9i} (1 - S_{W75i} )^{0.5} }}} \right)} \,\,$$

where N denotes the number of those samples with log density porosity greater than 9% and saturation of water less than 75%, *φ*_*n9i*_ denotes the neutron porosity of those samples having log density porosity greater than 9%, and S_w75i_ is the saturation of water of such samples.

#### Calibration of the seismic data

**Figure 5:**
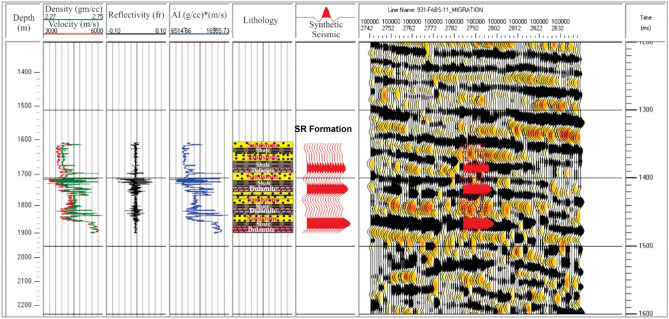
Synthetic seismogram (red traces) for the Bijnot-1 well (Salt Range Formation), showing from left to right: true vertical depth in meters, sonic velocity (µs/ft) and bulk density (g/cm^3^) logs, reflectivity (reflection coefficient), acoustic impedance (AI), lithology, synthetic seismogram, traces from a part of seismic line FABS-11, and two-way time in milliseconds.

Both the conventional logs (sonic and bulk density) and check shot data (time–depth relation) of the Bijnot-1 well along the seismic line were loaded into the computer program to calculate the synthetic seismogram. The acoustic impedance (AI) was obtained as the product of the sonic velocity (V_P_) and bulk density log, as given in Eq. (2). Using AI, the reflection coefficient (reflectivity) at each interface was then calculated by Eq. (3). In the process, we extracted the wavelet from the seismic dataset, specifically the seismic traces from the seismic data. The wavelet extraction derived from digitized AI and bulk density logs was convolved with the series of reflectivity to compute a synthetic seismogram. The reflectivity was converted from the depth domain to the time domain using available check shot data from wells. Finally, the synthetic seismogram was correlated with the seismic traces to build an impedance model. The AI curve closely matched the lithofacies (e.g., high AI is related to dolomite and shale). Mainly for this dataset, we selected the Ricker wavelet with a 2-ms sample rate, 25 Hz frequency, and 128-ms sample lengths to generate the synthetic trace. Substitutions such as sonic data calibration, datum, drift corrections, and seismic trace correlation were also performed during the construction of the synthetic seismogram. Figure 5 shows the synthetic seismogram, AI, reflectivity series, and the computed extracted wavelet for seismic line FABS-11.

2 $$\mathrm{AI }=\uprho \times {\mathrm{V}}_{\mathrm{P}}$$

3 $$\mathrm{RC }=\frac{ {(\mathrm{AI})}_{\mathrm{layer}-1}-{(\mathrm{AI})}_{\mathrm{layer}-2}}{{(\mathrm{AI})}_{\mathrm{layer}-1}+{(\mathrm{AI})}_{\mathrm{layer}-2}}$$

#### Seismic data inversion

We applied a joint inversion strategy in this work that incorporates MLC and PSO algorithms to convert seismic reflection data to spatial variability in the AI and MI profiles in the Infracambrian SR formation over the studied region^44,45^. Open-source rock star seismic inversion software (www.Chinarockstar.com) was used to predict the MI profile. Seismic line and MI data (input) were loaded into the inversion software, and the parameters according to the provisions of input data were adjusted to estimate MI (output).

#### Surface geochemical survey

Surface geomicrobial soil and soil gases were studied to explore the prospective areas of hydrocarbon microseepage and their origin. For this purpose, we collected 81 soil and gas samples along seismic lines over the study area, as shown in Fig. 1b. The samples were extracted from varying depths, 0.8 to 1.5 m, using a soil sampler, mechanical auger, sterilized soil sampling bags, and vacuum gas sampling bags. To evacuate the gas, a syringe was injected into the septum after sealing the annulus between the sampling tube and the borehole, as described by Tedesco^24^. While the area of interest was situated within the arid climate zone, all necessary measures were taken into consideration during sampling, e.g., avoiding sampling the soils from water-saturated as well as excavated areas and land contaminated by hydrocarbons, animal feces, the flow of chemicals, marshes, and the areas under the water table^21^.

We incubated soil samples with light hydrocarbon gases in the mineral salt medium for geomicrobial analysis and counted the hydrocarbon-oxidizing bacteria that had developed. For gas analysis, hydrocarbon gases desorbed from soil samples through acid–base extraction were analyzed by gas chromatography. The results are shown using concentration distribution maps to identify areas with anomalously high concentrations. Anomalies were marked through a statistical analysis approach, where the mean plus half of the standard deviation was assumed to be the background value.

## Result and interpretation

### Rock–Eval pyrolysis

**Figure 6:**
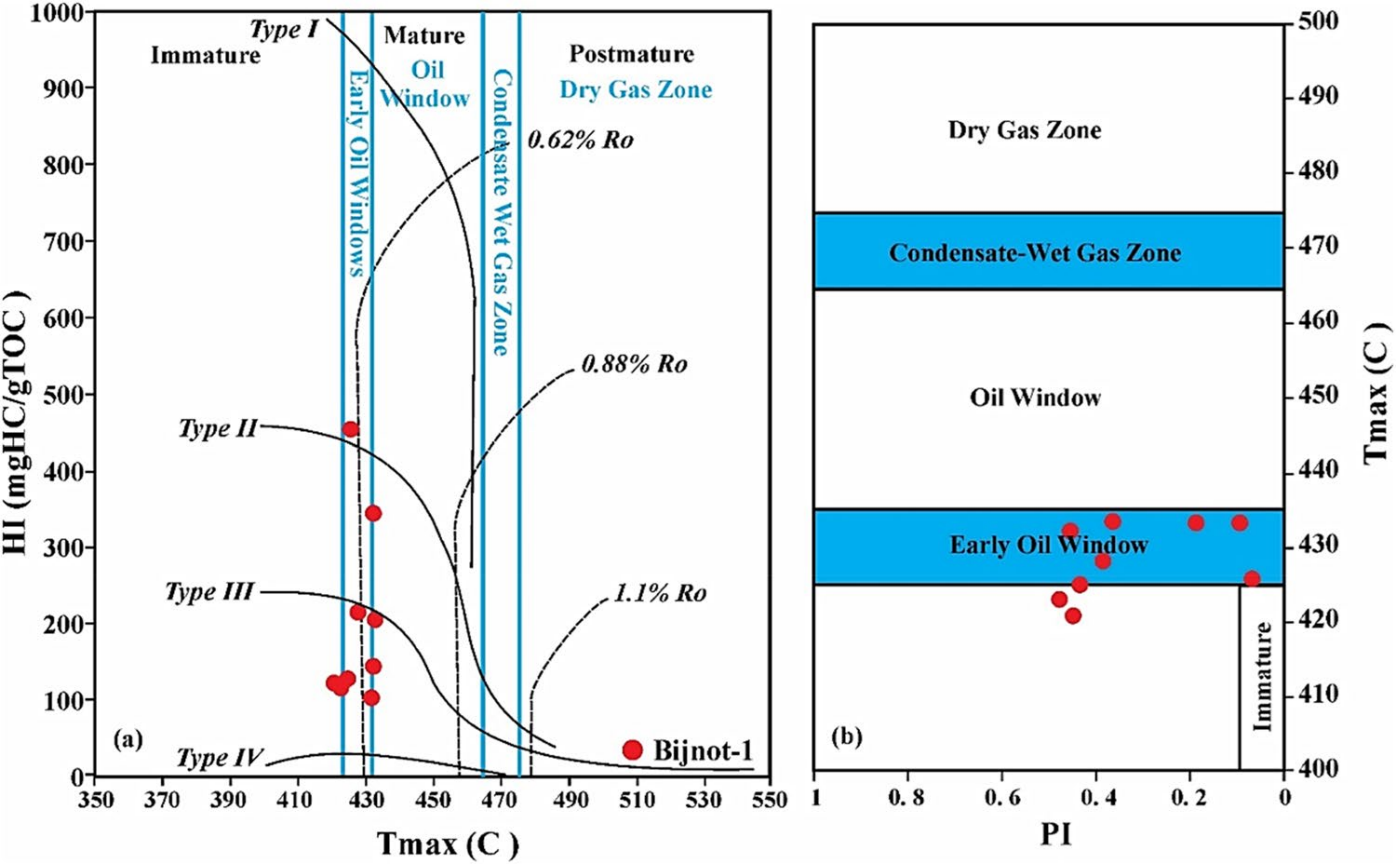
Thermal maturation assessment of the Infracambrian Salt Range Formation: (**a**) plot of HI versus T_max_, and (**b**) PI versus T_max_^39^.

The results of Rock–Eval pyrolysis of the Bijnot-1 well cuttings (Table 3) show TOC and HI values varying between 0.61 and 2.41 wt% with an average of 1.01 wt% and 104–451 mg HC/g TOC with a mean value of 209.7 mg HC/g TOC, respectively, in the Infracambrian SR Formation. The HI versus T_max_ plot reveals that more than half of the samples have reached an early mature stage of oil window generation. A mixture of types II and III is present in the SR Formation source rock with predominantly type III kerogen (Fig. 6a). This mixture with the predominance of type III explains the enrichment of the original OM in the oxygenated components and, consequently, the high values of OI. This OM could be cellulose and lignin consisting of high plants or ancestors, with a mixture of bacteria and phytoplankton source biota. In the PI versus T_max_ plot, the PI values fall between 0.08 to 0.46 and the T_max_ values range from 420 to 435 °C, further confirming that the Infracambrian SR Formation is thermally immature to early-mature (stage of oil generation window) (Fig. 6b).

### Burial history: maturation and hydrocarbon generation

#### Burial and thermal history modeling

The regional erosional unconformity between the Cretaceous and Paleocene, which is the most critical erosional surface in the study area, was considered when modeling the burial history. While developing the models, we assumed that the total erosion was between 40 and 180 m. However, we found that erosion had a negligible impact on present-day maturity and temperature trends after conducting comprehensive sensitivity analysis.

**Figure 7:**
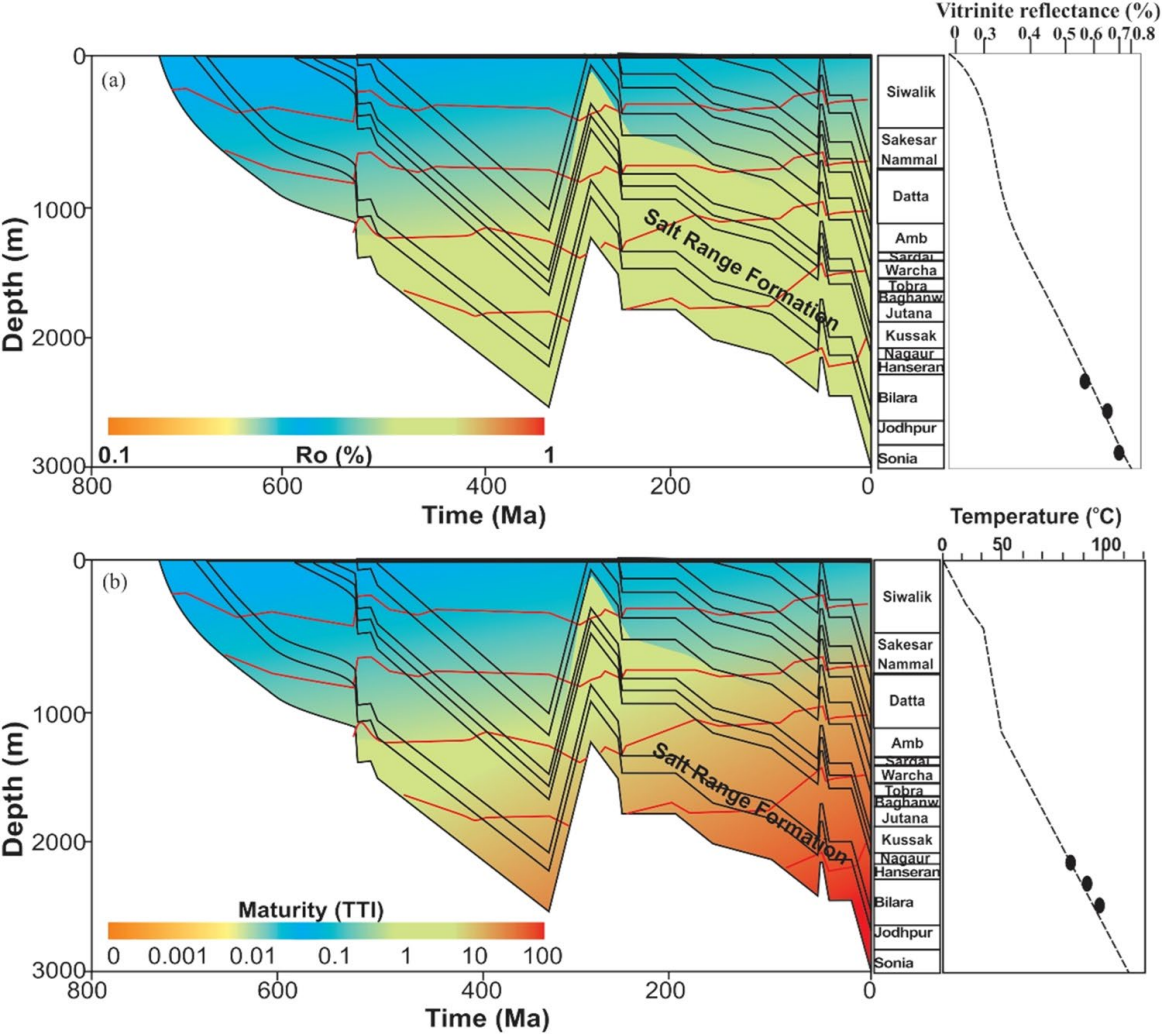
Burial history and thermal modeling of Infracambrian rock: (**a**) vitrinite reflectance, and (**b**) maturity profile.

In the Bijnot-1 well, a constant heat flow of approximately 72 mW/m^2^ results in the best calibration between the measured and calculated vitrinite reflectance. Burial history and thermal maturity modeling of Infracambrian rock demonstrated that Sonia, Jodhpur, Bilara, and Hanseran reached the early stages of the oil generation window, with a maximum burial temperature of approximately 102 °C and calculated vitrinite reflectance of 0.40 to 0.76% R_o_ (Fig. 7a,b).

#### Thermal maturity

For the maturity profile, the Infracambrian rock (Sonia, Jodhpur, Bilara, and Hanseran Evaporites) falls within the oil generation window. The present-day calculated time–temperature index (TTI) is 35. The vitrinite reflectance (R_o_) and temperature in the Infracambrian source rocks encountered in the Bijnot-1 well of SE Pakistan suggest an immature to early stage oil generation window (Fig. 7b).

### Biomarkers

#### Source rock depositional environment

**Figure 8:**
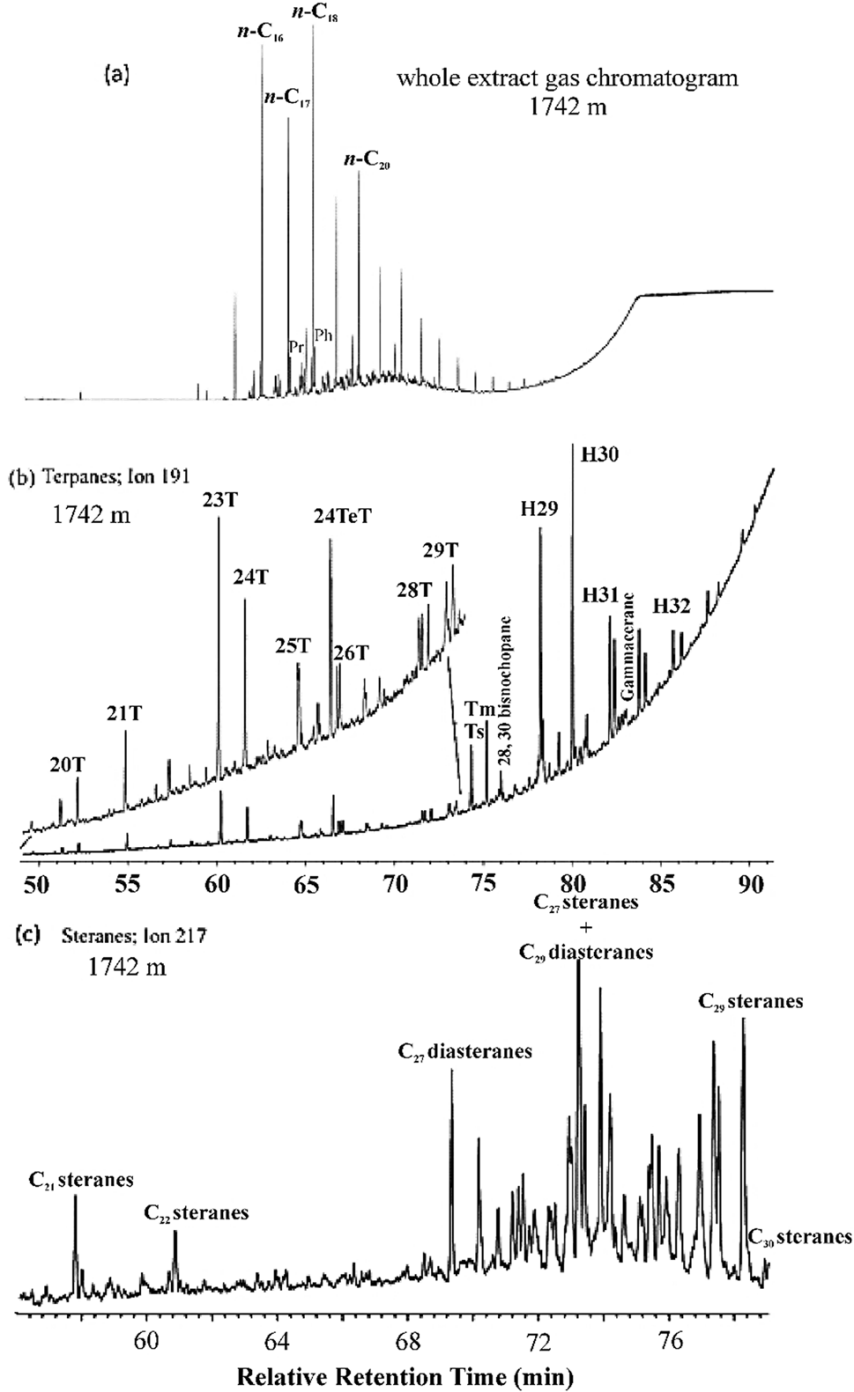
(**a**) Whole extract gas chromatogram of the Bijnot-1 sediment (1742 m) showing *n*-alkane and isoprenoid distributions, (**b**) selected ion chromatogram (191) showing the distribution of tricyclic and pentacyclic (Hopanes) terpanes in a representative sample from Bijnot-1. Letters with T and H refer to tricylic terpanes and hopanes, respectively, the numbers on the peaks represent the carbon numbers of the respective compound, and 24TeT indicates 24 tetracyclic terpane, and (**c**) selected ion chromatogram (217) showing the distribution of steranes and diasteranes. The numbers on the peaks represent the carbon numbers of steranes and diasteranes.

A representative sediment whole extract gas chromatogram (WEGC) is shown in Fig. 8a. The *n*-alkanes distribution profile shows a higher abundance of even-carbon number *n*-alkanes than odd-carbon number *n*-alkanes. This suggests a reducing environment of organic matter deposition where even-carbon number *n*-alkanes were preferably preserved compared to odd-carbon number *n*-alkanes^46^. An exclusive abundance of lower carbon range alkanes ~ C_16_–C_20_ in the WEGC suggests that marine organic matter dominates in the sediments^37,47^. Pristane/phytane (Pr/Ph) is a good indicator of OM depositional conditions^48^. The obtained Pr/Ph values were approximately 1 in the upper part of the analyzed interval and then decreased significantly with depth to relatively lower values (0.40) at the bottom (Table 4). These values suggest a suboxic environment of deposition in the upper part (1622–1634 m), while the middle section showed anoxic depositional conditions^40,41,49^.

**Table 4:** Biomarker ratios from the Infracambrian rock sequence of Bijnot-1, (Pr/Ph: pristane/phytane; Ts/(Ts + Tm), 18α(H)-22,29,30-trisnorneohopane/(18α(H)-22,29,30-trisnorneohopane + 17α(H)-22,29,30-trisnorhopane); C_32_ S/(S + R), C_32_ hopane, 22S/(22S + 22R), C_29_/C_30_ hopane: 17α(H),21β(H)-C_29_ hopane/17α(H), 21β(H)-C_30_ hopane; C_31_ R/C_30_ hopane, C_31_ αβ-homohopane (22R)/C_30_ αβ-hopane).

Sample	Depth (m)	Pr/Ph	C_32_ S/(S + R)	T_s_/(T_s_ + T_m_)	C_29_/C_30_ hopane	C_31_R/C_30_ hopane	DBT/phe-nanthrene
BJ-01	1622	1.08	0.63	0.45	0.76	0.33	0.13
BJ-02	1634	1.18	0.65	0.11	0.70	1.80	0.30
BJ-04	1642	0.93	0.63	0.39	0.78	1.19	0.22
BJ-05	1662	0.61	0.63	0.45	0.80	0.22	0.13
BJ-06	1682	1.03	0.58	0.32	0.61	0.32	0.14
BJ-08	1700	0.67	0.61	0.38	0.75	0.36	0.10
BJ-09	1742	0.80	0.59	0.44	0.74	0.29	0.14
BJ-10	1762	0.49	0.65	0.42	0.84	0.39	0.13
BJ-12	1794	0.40	0.58	0.43	0.70	0.31	0.18

#### Possible source facies

The distribution of tricyclic terpanes, steranes, and hopanes in the cutting extracts (1742 m) was applied to determine the variation in source facies in the SR Formation (Fig. 8b,c). Hopanes are present in higher relative abundance than tricyclic terpanes. The C_23_ compound is the predominant peak among the tricyclic terpanes and is particularly abundant compared to the C_24_ tetracyclic compound. The C_23_/C_24_ ratio is approximately 1.5. This ratio reflects the importance of tricyclic terpanes with regard to tetracyclic terpanes and is significant for the abundance of the precursor of these compounds (tricyclohexaprenol) synthesized by bacteria as a stabilizer of microorganism membranes^17^. The tricyclic terpanes are known as "orphan biomarkers", as their precise sources are unknown. In fact, these could be derivatives of algae. This indicates that the OM of the SR Formation source rock could have originated from a marine source facies enriched with microorganisms. In addition, the presence of C_24_ at small but significant concentrations reflects a possible minor contribution of terrestrial OM sources. Peters and Moldowan^50^ found that the C_24_ tricyclic terpane is related to source rocks containing continental organic matter in respectable amounts. Additionally, C_20_ and C_21_ are relatively abundant, and C_20_ tricyclic terpane abundance is related to terrestrial plant sources. The abundances of C_25_ and C_26_ hopanes are similar, with C_26_/C_25_ tricyclic terpane ratios less than 0.9^17^, indicating a prevalent marine deposit environment. Similarly, the C_24_ tetracyclic terpane is more abundant than the C_26_ tricyclic terpanes at almost all depths, revealing marine carbonates to marl facies.

Stable 17α(H)-, 18α(H)-, 21β(H)-28-, and 30-bisnorhopane was detected at low concentrations compared to the hopane ratios. This confirms that the depositional environments in SE Pakistan during the Infracambrian were generally suboxic. In contrast, the high C_28_ bisnorhopane is related to reducing physicochemical conditions in organic matter deposition environments^51^. For the pentacyclic terpanes, the C_29_ and C_30_ hopane contents are relatively high. The C_29_/C_30_ hopane ratio has been used to differentiate shale- and carbonate-sourced oils^50,52^. The C_29_/C_30_ hopane ratios are relatively high in carbonate- and evaporate-sourced oils (~ 0.7 or greater) and lower for shale-source oil (0.4–0.75). However, this ratio is influenced by thermal maturity. In our analysis, the C_29_/C_30_ hopane ratio varies from 0.61 to 0.84, indicating carbonate source rock. Gammacerane is present in low concentrations, which indicates a stratified water column induced by salinity differences. It is commonly considered of tetrahymanol origin, which is a chemical that occurs in saline waters^53^. The C_31_R/C_30_ ratio is generally used to distinguish between lacustrine and marine depositional environments, and sediments with a ratio of less than 0.25 are interpreted as lacustrine deposits^17^. The hopane versus homohopane ratio (C_31_R/C_30_) of the SR Formation samples is more than 0.25, indicating a marine source facies origin.

We observed that a series of C_27_–C_29_ steranes were abundant in the samples, with C_27_ and C_29_ steranes predominating over C_28_ steranes. Additionally, we noted that diasteranes were present in moderate to high proportions and were less abundant than regular steranes, especially the C_27_ diasteranes. This observation indicates a clay-starved carbonate to marl source facies in the SR Formation throughout the analyzed section. The relative proportions of C_27_ and C_29_ have been proposed to indicate the origin of organic matter. The C_29_ and C_27_ steranes are derived from terrestrial and marine sources, respectively. The presence of C_27_ steranes in high amounts compared to C_29_ in the SR Formation proves the marine depositional environment of the OM. The significant amounts of C_29_ steranes present are derived from marine green algae, the ancestors of terrestrial land plants. In fact, life during the late Neoproterozoic-early Cambrian was restricted to the marine realm, thus ruling out the terrestrial OM contribution^54^. The C_30_ steranes were present in small but significant amounts. These biomarker features indicate a marine OM associated with marly limestone source rock deposited in suboxic and normal salinity depositional marine environments. The biomarker features of the SR Formation showed a slight variation in the source facies within the section. The upper part has comparatively more carbonate content than the lower section. The abundance of a sulfur-bearing heterocyclic aromatic hydrocarbon, dibenzothiophene (DBT), is an excellent source facies indicator compared with that of other aromatic hydrocarbons^55^. The DBT/phenanthrene ratios from the Bijnot-1 sediments reached up to 0.3, indicating the significant presence of sulfur aromatics, a common phenomenon of marly source facies. These results are consistent with a deep-water marine depositional environment with restricted conditions suitable for carbonate sediments^31,56^.

#### Thermal maturity

Aliphatic and aromatic hydrocarbon parameters were applied to evaluate the thermal maturity of the SR Formation sediments. Isomerization at the C-22 positions in C_31_ to C_35_ 17α(H)-hopanes^57^ occurs prior to most biomarker reactions and is used for thermal maturity assessment as isomerization at the C_20_ positions in regular steranes. Additionally, important isomerization reactions during thermal maturation occur at C-17 and C-21. Similarly, important isomerization reactions occur at C-14 and C-17 for steranes. In response to temperature augmentation, the biological 22R configuration transforms to a 22R and 22S mixture. As a result, the 22S/(22R + 22S) homohopane ratio could generally be used for determining maturity. However, homohopane isomerization attains equilibrium at the early maturity stage; therefore, the 22S/(22R + 22S) homohopane ratio cannot be used to determine higher-maturity stages^42^. In the analyzed OM, the C22S/(22S + 22R) hopane values ranged from 0.58 to 0.65 without a significant difference in the values for the analyzed section, showing that the isomerization had attained equilibrium and the SR Formation sediments were at the early mature stage to intermediate maturity.

On the other hand, the Ts/(Ts + Tm) ratio shows values of approximately 0.35, indicating that the OM has not yet reached the oil generation window, given that Ts is more stable regarding thermal maturity than Tm and the ratio Ts/(Ts + Tm) increases with increasing maturity. The results for these immature Precambrian rocks are consistent with those from previous studies of the basin^49^. However, if we examine the rest of the thermal maturity biomarker parameters of the samples from the SR Formation, especially the deeper ones, we see a gradual increase in thermal maturity. Pregnane (C_21_-sterane) and methyl-pregnane (C_22_-sterane) are also used as thermal maturity biomarkers since they are characteristic of light oils^58,59^. In the laboratory, these steranes are produced by thermal cracking at 300 °C for a long period^58^. In the OM of the SR Formation, C_21_ and C_22_ steranes were detected in high concentrations (Fig. 8c). These pregnanes are a good indicator of relative thermal maturity (end of s.s. diagenesis-beginning of catagenesis) and the alteration of OM in SE Pakistan. Aromatic hydrocarbon maturity parameters from alkylphenanthrene and alkyldibezothiophenes also support the maturity evaluation of the early stage oil generation window. Additionally, the results of Rock–Eval pyrolysis (T_max_ > 430 °C) support the early-mature stage of the oil generation window in the Infracambrian SR Formation. In addition, the results of geochemical surface exploration highlight the presence of thermogenic C_2_–C_4_ hydrocarbons, supporting the mature stage of the source rock in the subsurface.

Overall, the biomarker features of SR Formation show a slight variation throughout the analyzed section. These variations in parameters most likely reflect the subtle differences in the sedimentary facies, lithology, depositional environment, and conditions. The upper part has more carbonate content deposited in the marine environment under suboxic conditions (C_31_R/C_30_ values of 1.8 and 1.19 at depths 1634 m and 1642 m, respectively; Pr/Ph values near 1 and Ts/(Ts + Tm) = 0.11 at 1634 m) compared to the lower section, which is more mature and deposited under anoxic conditions, as proven from the higher Ts/(Ts + Tm) ratio and lower Pr/Ph ratio, respectively.

### Application of ML algorithms for seismic data inversion

#### AI inversion

**Figure 9:**
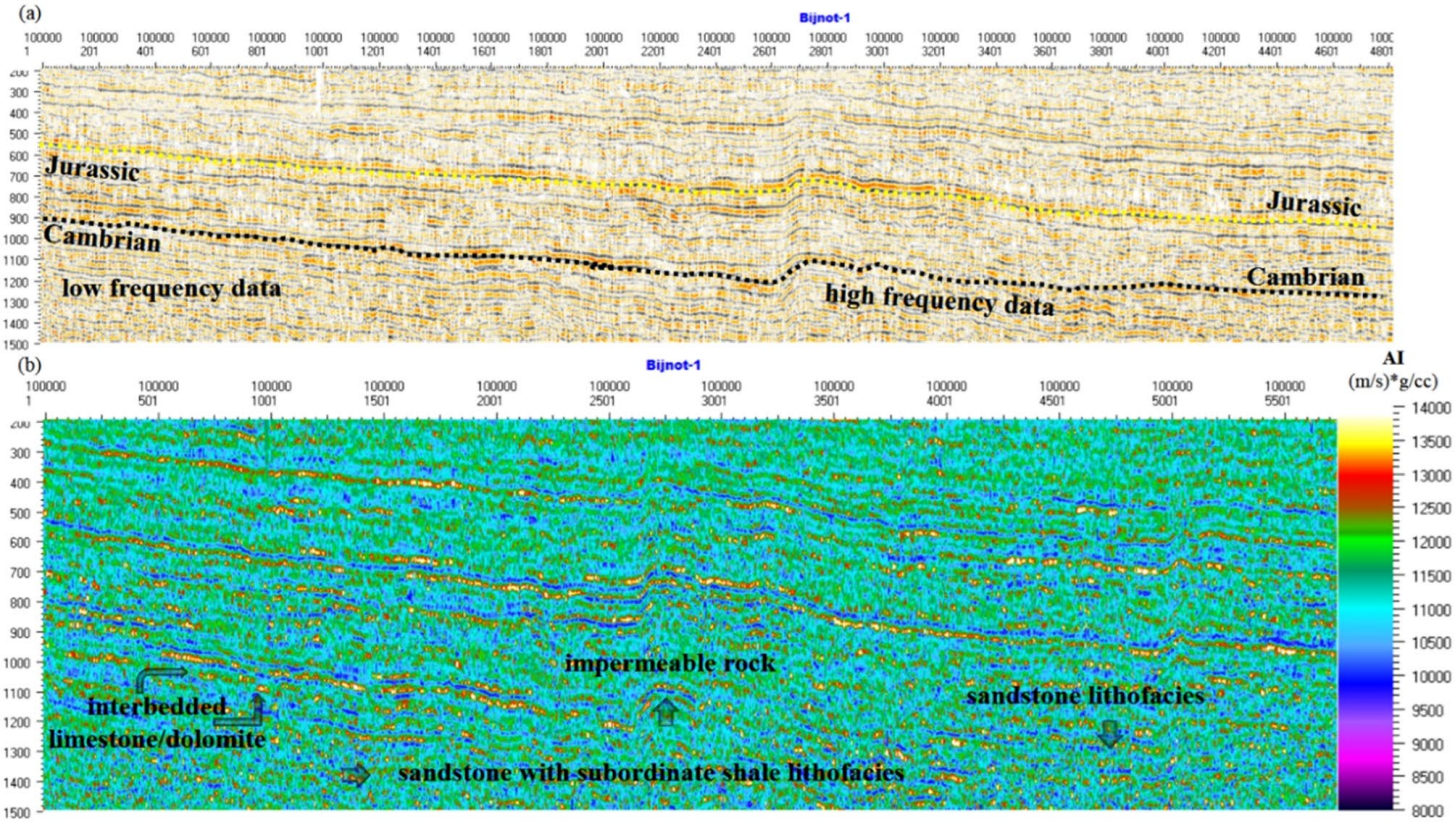
(**a**) Interpreted seismic line of FABS-11, and (**b**) the relative inverted acoustic impedance using the MLC and PSO inversion strategy.

AI inversion transforms seismic data into pseudoacoustic impedance logs at every trace. Figure 9a,b shows the interpreted seismic line of FABS-11 and inverted AI using the MLC and PSO inversion strategy. It has been demonstrated that the AI in the zone of interest, i.e., Cambrian and Infracambrian sequences (900 to 1460 ms), varies from 9000 to 13,500 (m/s) × (g/cc). These variations in AI are associated with significant lithological changes to sandstone, shale, limestone, dolomite, and sandstone with subordinate shale lithofacies. It should be noted that the low AI (9000–11,500 (m/s) × (g/cc)) between the time intervals of 1250 to 1450 ms perhaps indicates a potential hydrocarbon-bearing zone at this particular interval (arrow). However, the overlying red–yellow horizons just above the blue horizons (arrow) reveal higher values of AI (12,500–13,500 (m/s) × (g/cc)) and are, hence, most likely an impermeable seal rock for a potential reservoir. Harder rocks, i.e., compact limestone, have higher AI values than sandstone and clay^44^. Note that irregular patterns of low and high AI reflecting interbedded limestone and dolomite layers are well captured by the MLC and PSO inversion strategies (indicated with arrows) (Fig. 9b). Since a nonlinear relationship exists between the interbedded thin layers and seismic waveforms, PSO can provide a nonlinear projection correlation to determine the interbedded layers from the seismic waveform^60^. Good reservoir quality areas and thin layer beds clearly appear in the Cambrian and Infracambrian intervals (arrow).

#### MI prediction

**Figure 10:**
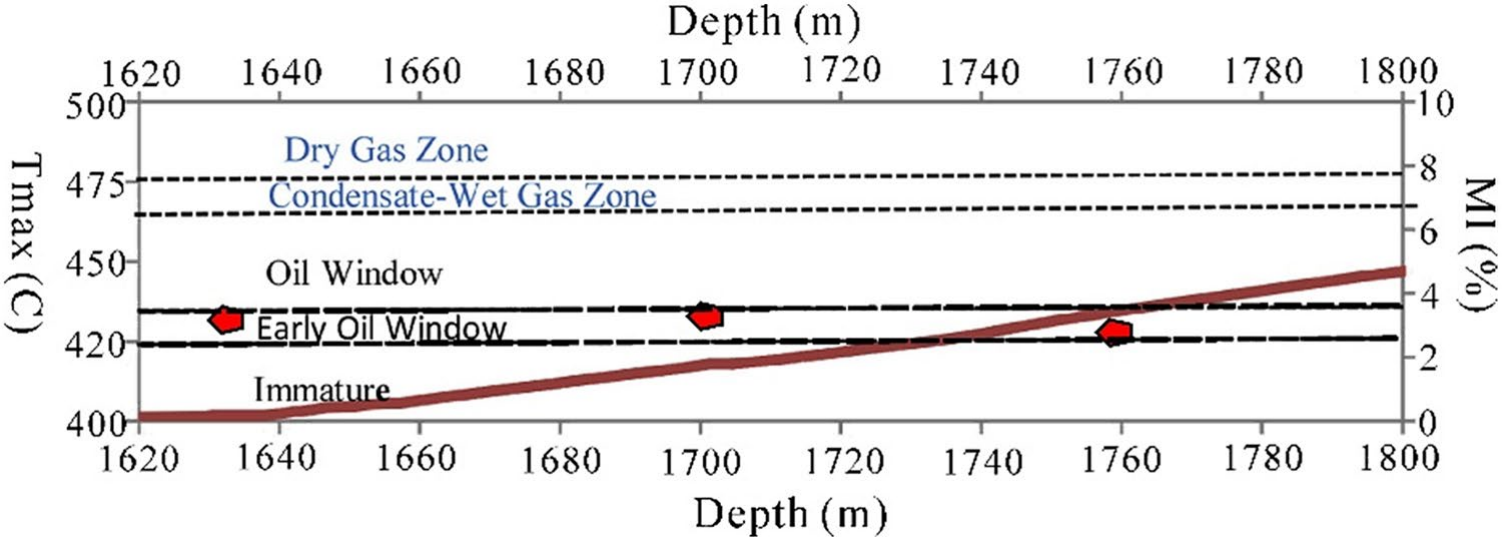
Graphical comparison between the calculated maturity index (MI) and T_max_ in Bijnot-1 (red dots represent T_max_ and maroon line for MI).

MI is an essential parameter for determining the thermal maturity levels of hydrocarbon source rock and correlating them with the thermal maturity evaluated from geochemical methods. Figure 10 shows a graphical comparison between the calculated MI using Eq. (1) and the measured T_max_ of the Infracambrian SR Formation in Bijnot-1. The measured T_max_ (red dots) falls within the early stage of the oil generation window in the range of 420–435 °C with an MI (maroon line) of 2–3%.

**Figure 11:**
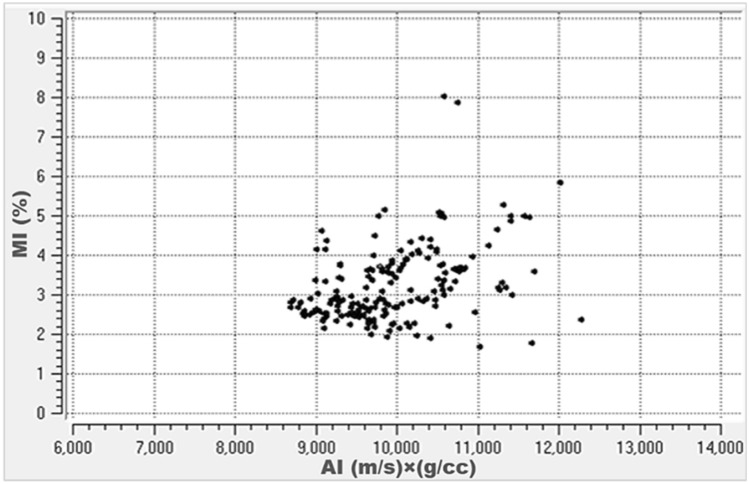
Maturity index (MI) versus acoustic impedance (AI) cross-plot.

Figure 11 shows a cross-plot of MI and AI. The plot shows a positive linear correlation between MI and AI. MI increases with increasing AI.

**Figure 12:**
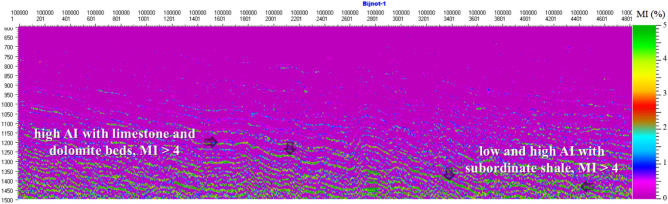
Seismic inverted MI profile using the MLC and PSO inversion strategy in Cambrian and Infracambrian rock sequences at 1100–1500 ms.

In Fig. 12, the inverted AI surface is converted into an inversion solution for an MI map using the MLC and PSO inversion strategy^43,61^. It is shown that the deeper horizons reveal fair to good MI values, i.e., 3 to 5% with low AI, and correspond to good reservoir quality areas (arrow). It should be noted that blue and sky blue layers in the middle and upper parts of the profile with MI < 2 correspond to the immature oil generation stage, whereas yellow and green layers in the lower part of the profile with MI > 4 correspond to the early oil generation stage. The results of MI are in agreement with the earlier research of Abdizadeh et al.^61^ and correlate well with the thermal maturity measured from Rock–Eval pyrolysis and biomarkers in the studied interval. Generally, thermal maturity and MI increase as a function of depth; however, structural features such as structural highs and lows, salt-induced anticlines, and paleohighs could produce local thermal anomalies.

## Discussion

### Surface geochemical and geomicrobial surveys

**Figure 13:**
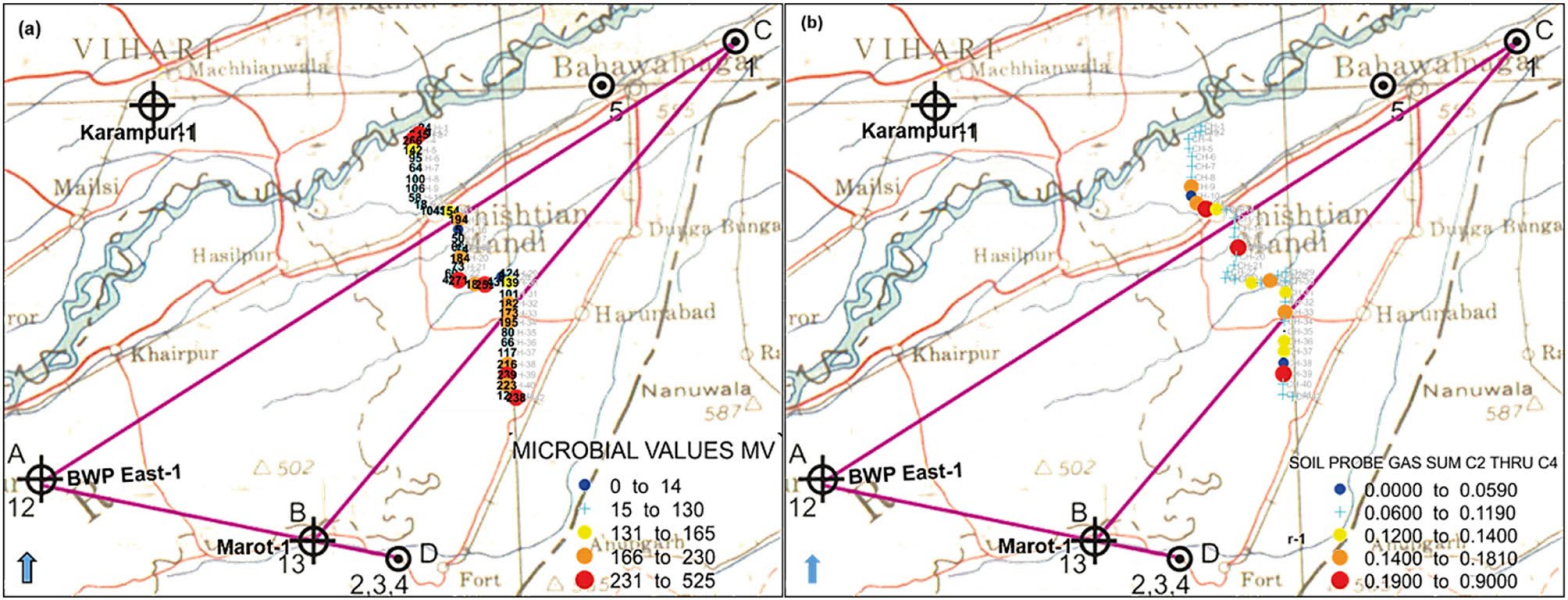
(**a**) Distribution plot of microbial value anomalies, and (**b**) distribution plot of soil probe C_2_–C_4_ hydrocarbons. Microbial and soil probe hydrocarbon values overlap with toposheets of the study area.

The abundance of hydrocarbon-oxidizing bacteria ranged from 0 to 525 × 103 (cfu/g of soil sample) (Fig. 13a). The hydrocarbon gases measured by gas chromatography show concentrations of C_2_ through C_4_ ranging from 0 to 900 ppb (Fig. 13b).

The geomicrobial and soil gas concentration distribution maps demonstrate that the anomalies coincide and are significantly inversely correlated. In fact, hydrocarbon microseepage provides optimal conditions for developing highly specialized microbial populations that depend solely on light hydrocarbon gas^21^. As a result, these microbes grow at abnormally high concentrations in near-surface soils and geological structures that contain hydrocarbons. It is worth noting that the obtained results represent the normal distributions of gas and microbial concentrations. This inverse correlation is caused by bacteria that oxidize hydrocarbons consuming soil gas. Light hydrocarbon microseepage is consumed, particularly in the highest seepage area, allowing for a high bacterial activity rate and hydrocarbon gas concentration depletion. The bacteria located directly above the flow chimney would transform the petroleum gases, while the edges would have large gas concentrations as they are unutilized by bacteria and where all microbial activity is prolonged.

The soil probe gas analyses show variable concentrations where C_1_ is absent, and the samples contain C_2_–C_4_ hydrocarbons. However, the amounts of migrated gases usually decrease in the order of methane > ethane > propane > butane. We can attribute the deficiency of methane to its total consumption by the methanotrophic bacteria in the soil. Because it is the simplest hydrocarbon molecule, methane is the first and easiest component to decompose by bacteria, causing its complete depletion in the soil gas samples in proportion to the other components. A previous study conducted in the adjacent Bikaner–Nagaur Basin highlighted the presence of anomalous populations of n-pentane- and n-hexane-oxidizing bacteria in surface soils^62^.

The presence of heavier components of hydrocarbon gases, mainly propane and butane, favors a thermogenic origin of the detected gases. Nevertheless, another source of hydrocarbon gases could be detected on the surface, such as that of biogenic origin.

**Figure 14:**
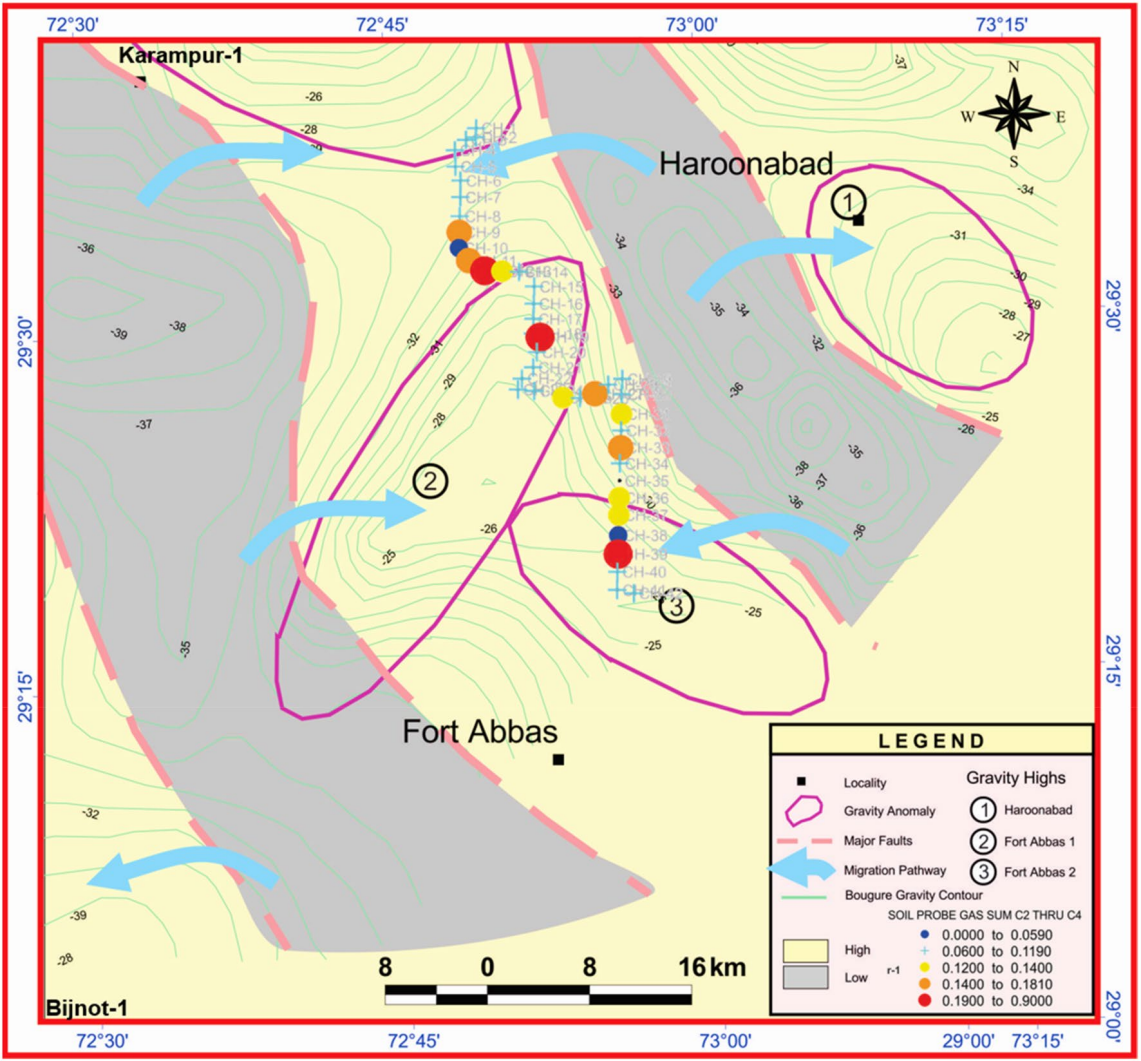
Overlapping C_2_–C_4_ hydrocarbon anomalies on the interpreted gravity anomaly map. Surfer (www.goldensoftware.com) and CorelDRAW X5 (www.coreldraw.com) were used to create the illustration.

To further investigate the origin of these surface gases, we included other studies, such as geophysical characterization, including gravity modeling and seismic data interpretation, to explore if possible deep pathways exist, in addition to maturity estimation and biomarker analysis. The soil probe gas concentrations are plotted and overlapped on the gravity anomaly map (also ties with seismic data interpretation and the geological cross-section). The composite map of the C_2_–C_4_ hydrocarbons and gravity anomalies shows a good correlation between high concentrations of C_2_–C_4_ hydrocarbons and the zone of gravity high, as well as major faults (Fig. 14). The C_2_–C_4_ hydrocarbons show high concentrations along the faults separating gravity highs and gravity lows.

The seismic lines along the surface geochemical study were also interpreted to show the structural features and possible migration pathways. The stratigraphic horizons were marked and tied using check shot velocities. The cross-sections along the seismic lines GG′ (north to south) and II′ (east to west) were prepared and converted to depth. We note that salt-induced structural leads identified at the Infracambrian level show a continuous rise from the basement overlying stratigraphic horizons from west to east.

**Figure 15:**
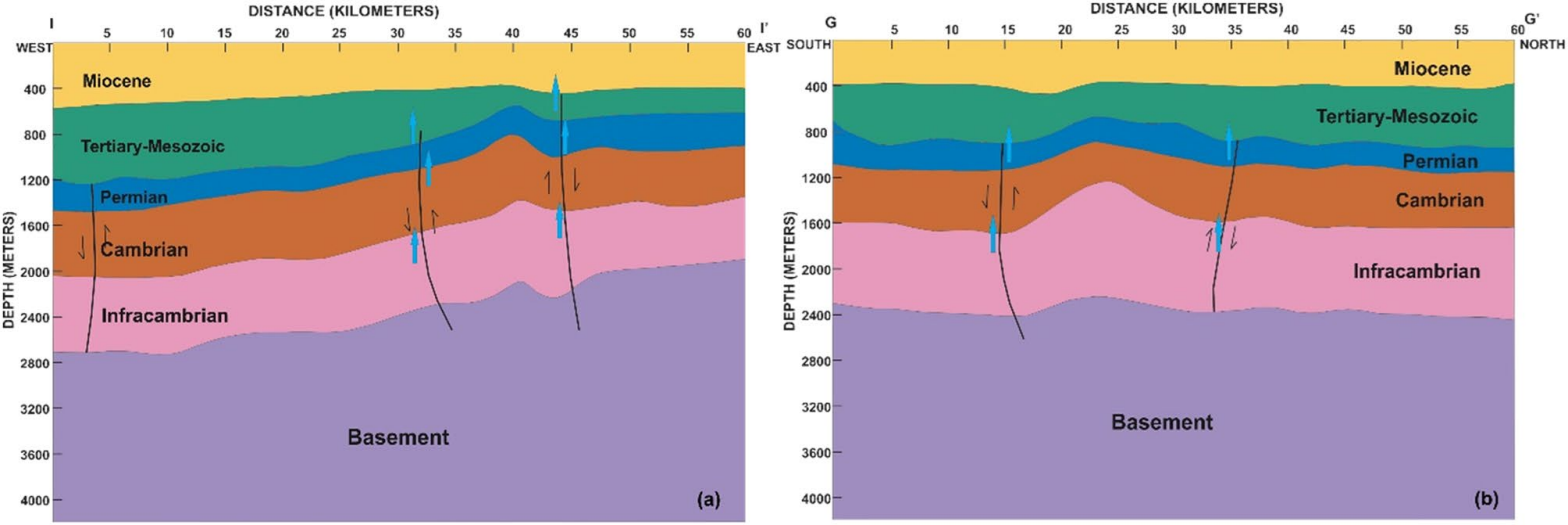
Seismic converted depth section indicating seepage through the main faults (shown with blue arrow): (**a**) along cross-section II′, and (**b**) along cross-section GG′.

A geoseismic depth section along line II' reveals a basement high located at a distance of 40 km (Fig. 15a). The overlying Mesozoic–Palaeogene sequence follows the rise in the basement. A four-way structural closure of 80 ms (~ 160 m) (vertical closure) is mapped in the eastern part of the study area. A normal fault can also be observed in the seismic section. The Infracambrian top in this seismic section is identified at a depth of 1600 to 2200 m from east to west.

A geo-seismic cross-section with GG` is oriented in the NS direction (strike line). The cross-section shows that the basement starts rising from a distance of 15 km to the highest crustal part located at 25 km (Fig. 15b). The Infracambrian to Paleogene overlying the bulge in the basement shows prominent anticlinal four-way structural closure (as shown by the dip line from east to west). The thickness of the stratigraphic horizons remains constant. The Infracambrian top of this seismic section is identified at a depth of 1400 to 1600 m from north to south.

We concluded that deep-seated faults overlying the basement high as well as near-surface diffusion provided pathways for C_2_–C_4_ hydrocarbon migration to the surface, supporting the deep thermogenic origin of these gases. Moreover, this indicates the presence of an active petroleum system beneath the Earth's surface. In fact, the kerogen is subjected to increasing pressure and temperature during burial. Under these conditions, it undergoes thermal cracking, during which the kerogen's macromolecular network breaks up, releasing liquid or gaseous compounds. The quantity of bitumen increases as the proportion of kerogen decreases. There are three stages to this process. Most of the oxygenated activities are lost during the first stage, and carbon dioxide and water are produced. This first stage is known as diagenesis. It is represented on Van Krevelen's diagram by a decrease in the O/C ratio without a significant change in the H/C ratio. The second stage is catagenesis, or hydrocarbon formation, in which thermal cracking breaks the C–C bonds of hydrocarbon chains. As a result, the H/C ratio gradually decreases. At this stage, the temperature reaches 140 °C at depths ranging from 2000 to 3000 m. Most of the kerogen (50 to 90% by mass) is converted into fluid products (mainly hydrocarbons). Finally, in the third stage, there is a significant production of methane from the residual kerogen as well as the oil generated in the previous stage. These are some of the characteristics of thermogenic hydrocarbon gases.

### Regional correlation of Neoproterozoic rocks

**Figure 16:**
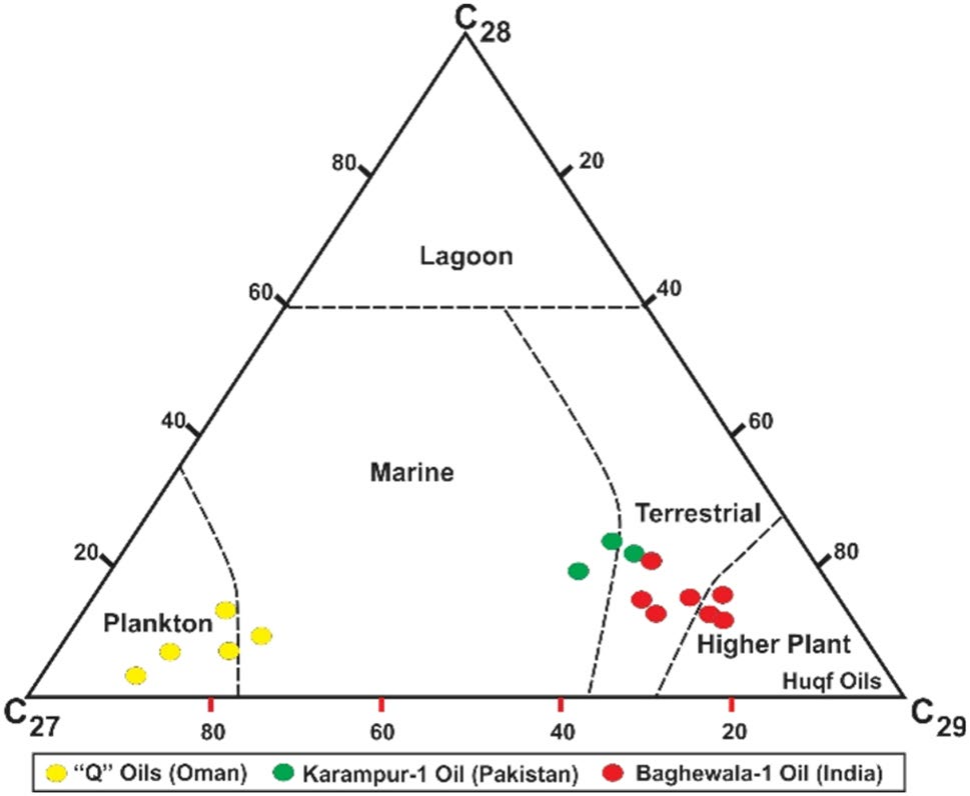
Analysis of the geochemistry of hydrocarbons (oil) in southern Oman 'Huqf' and 'Q' oil, Karampur-1 (Pakistan), and Baghewala-1 (India).

A literature study involving works from various authors revealed that the heavy oil shows from the Bijnot-1, Fort Abbas-1, Karampur-1, and Marot-1 exploratory wells are remarkably similar to those of the Baghewala-1 oil BK-N basin in India and southern Oman^2,9,12,63–65^. The heavy oil from the Bilara and Jodhpur formations in the Baghewala-1 well and the SR Formation in the Karampur-1 well geochemically correlate with oil on the eastern flank of the Infracambrian Huqf Group (Oman)^2,14,66^. This relationship is consistent with the plate tectonic reconstruction model. During the late Precambrian-early Cambrian period, marine evaporite deposits in northwest India, SE Pakistan, and southern Oman were close to each other on a large carbonate margin shelf of Gondwanaland^1,3,4,63,67,68^. Geochemical analysis (high proportion of C_29_ steranes, monoaromatic and diasterane steroids, low pristane/phytane ratio, low concentration of diasteranes, low API gravity, and high concentration of sulfur) of Karampur-1, Baghewala-1, and southern Oman suggest similar environmental conditions with the same age, as shown in Fig. 16.

**Figure 17:**
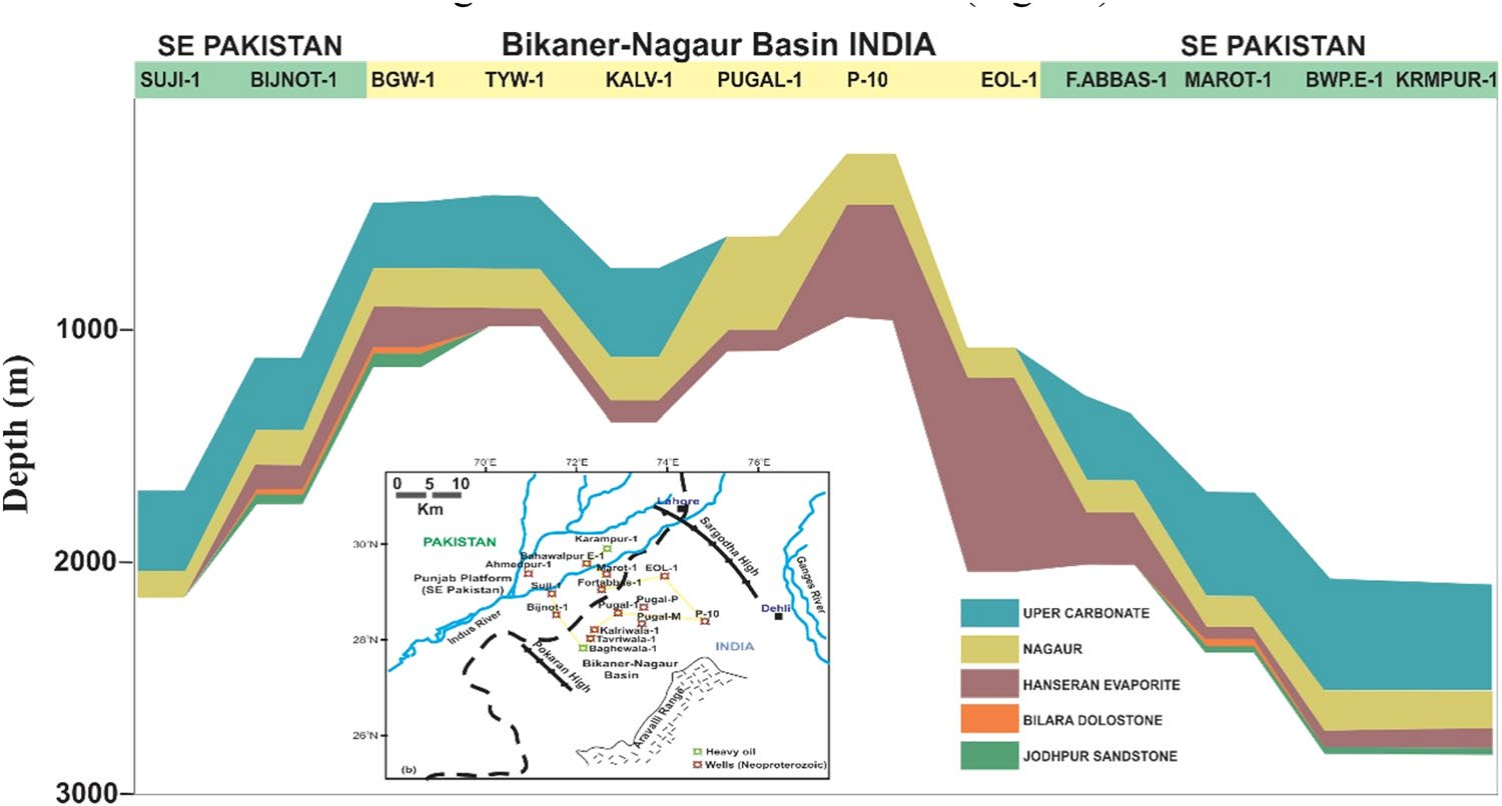
Subsurface correlation of Neoproterozoic sequences (the Nagaur, Hanseran, Bilara, and Jodhpur Formations) in SE Pakistan and the BK–N Basin in India.

Geological and geochemical correlations between Baghewala-1 and Karampur-1 favor the possibility of heavy and light oil reserves in deeper buried Infracambrian source rocks in the SE flank of Pakistan extending to the BK-N Basin of India (Fig. 17).

**Figure 18:**
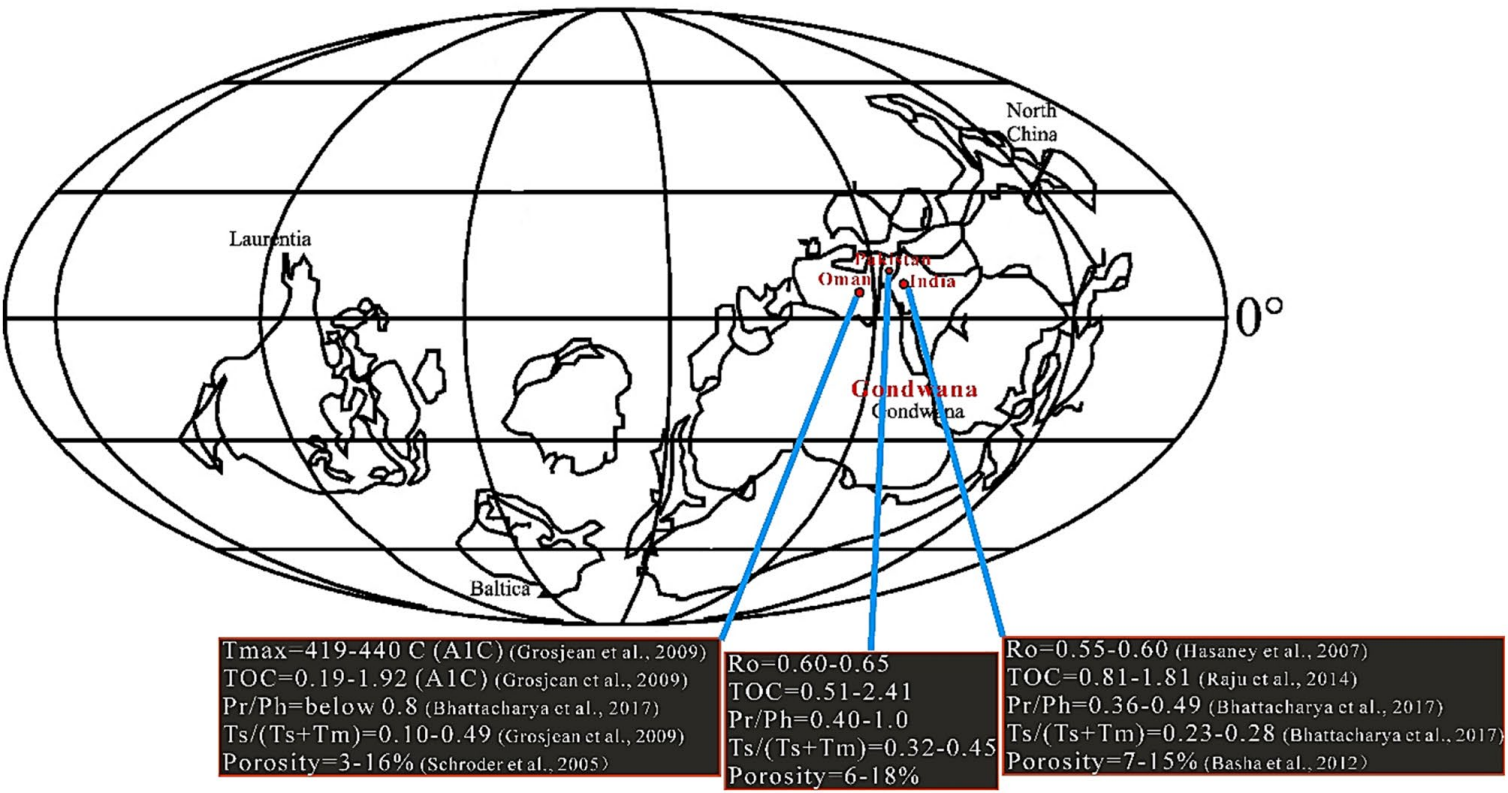
Distribution of various source and reservoir rock properties along with certain biomarker parameters in the Infracambrian rock sequence from SE Pakistan, India, and southern Oman. CorelDRAW X5 was used to create the illustration (www.coreldraw.com).

We investigated the potential and maturity of the Infracambrian source rocks in SE Pakistan based on integrated geochemical, geological, and geophysical data from soil and core samples. Rock–Eval pyrolysis and biomarkers identified marine restricted clay-starved carbonate as source rocks that have just reached the oil generation window and a mixture of type II and III kerogen. These kerogens have been expelled using several fault-controlled horsts and grabens and salt plug anticlinal structures in the overlying Infracambrian to Permian strata. The distribution of various source and reservoir rock parameters and specific biomarker parameters of the Infracambrian rocks from Pakistan are correlated with Neoproterozoic-early Cambrian successions from India and Oman Huqf oils (typified by the A1C oil) (Fig. 18). The distributions of TOC, vitrinite reflectance, and porosity are closely comparable. The Pr/Ph and Ts/(Ts + Tm) distributions in Baghewala-1 and Oman Huqf oils are similar, but Infracambrian rocks in SE Pakistan have a somewhat higher abundance of Pr/Ph and lower Ts/(Ts + Tm) with a slight variation throughout the analyzed section. These variations in parameters most likely reflect the differences in sedimentary facies, lithology, deposition conditions, and maturity. Due to localized uplift and a global eustatic fall in sea level, we can attribute these variations to the widespread Early-Middle Cambrian and the Middle/Upper Permian unconformity in the study area. The upper part of the SR Formation is sometimes absent due to erosion during the Cambrian and Permian unconformities, which could explain why the upper part is more oxygenated^10,11^. It is uplifted and, as a result, more exposed. This exposure makes the OM in the upper part subject to alteration and explains the immature biomarker features we detected in some samples and the possible minor terrestrial provision recorded.

## Conclusions

We documented the evaluation of source parameters from Neoproterozoic rocks (Infracambrian rock sequence) in SE Pakistan and adjacent BK-N Basin India using geophysical data, cores, Rock–Eval pyrolysis, biomarkers, and surface geochemical samples. Based on available data and techniques, we can draw the following conclusions:

1. The seismic and well log data interpretation identified the Infracambrian rock sequence at 1600 to 2200 m (east to west) with promising structural features. The structural interpretation of the geoseismic cross-sections and gravity anomaly maps show several fault-controlled horsts and grabens and salt plug anticlinal structures in the overlying Infracambrian to Permian strata.
2. From the investigated surface geochemical samples, anomalous concentrations of thermogenic C_2_–C_4_ hydrocarbons in surface soils above anticlinal structures confirmed the presence of a mature source rock capable of generating and expelling hydrocarbons. An effective migration is conveying those hydrocarbons along deep-seated faults to the surface.
3. The investigated core samples from the Infracambrian rock sequence using Rock–Eval pyrolysis (TOC = 0.61–2.41 wt% and HI = 104–451 mg HC/g TOC) and biomarkers (P_r_/P_h_ = 0.40–1 and T_S_/(T_S_ + Tm) = 0.32–0.45) show that deeper buried source rocks in the SE flank of Pakistan have marine restricted clay devoid of carbonate to marl source facies with thermal maturity (R_o_ = 0.60–0.65) in the early stage of the oil generation window and a mixture of type II and III kerogen.
4. An excellent correlation of thermal maturity deduced from the inverted MI profile with the results of the surface geochemical survey, source biomarkers, and Rock–Eval pyrolysis proved the reliability of the proposed MLC and PSO algorithms. The spatial variations in the inverted MI profile showed a reasonable estimation of thermal maturity for identifying the prospective areas and migration routes in the SR Formation.
5. The integration of surface geochemical surveys, source biomarkers, Rock–Eval pyrolysis, and geophysical characterization suggests that significant heavy and light oil reserves could be explored along the SE flank of Pakistan, where the Infracambrian rock sequence is thicker and thermally mature compared to that in the BK–N Basin, India, as well as the existence of deep-seated structural closure. However, estimating those potential reserves still requires more profound geophysical studies to validate the structural traps and related petroleum systems.

Insufficient support of geological and geophysical data from the SE flank of Pakistan hinders us from drawing a general conclusion for the distribution and potential of the Infracambrian reservoir in the basin. Therefore, more studies are still necessary to validate the structural traps and relative petroleum systems by interpreting high-resolution seismic and geochemical data for further screening of the conflicting maturity signatures.

## Data Availability

The datasets generated and/or analysed during the current study are not publicly available due to seismic and well logs data confidentiality but are available from the corresponding author on reasonable request.
